# Absence of NMDA receptor antibodies in the rare association between Type 1 Narcolepsy and Psychosis

**DOI:** 10.1038/srep25230

**Published:** 2016-05-04

**Authors:** Y. Dauvilliers, C. Gaig, L. Barateau, F. Graus, A. Iranzo, R. Lopez, J. Santamaria

**Affiliations:** 1 National Reference Centre for Narcolepsy, Sleep Unit, Department of Neurology, Hôpital Gui-de-Chauliac, CHU Montpellier, France; 2 Inserm, U1061, Université Montpellier 1, Montpellier, France; 3 Institut d’Investigacions Biomèdiques August Pi i Sunyer (IDIBAPS), Barcelona, Spain; 4 Department of Neurology, Barcelona, Spain; 5 Multidisciplinary Sleep Disorders Unit, Barcelona, Spain

## Abstract

Frequency and mechanisms underlying the association between narcolepsy type 1 (NT1) and psychosis remain unclear with potential role for a common immune pathway. We estimated the frequency of psychosis and its characteristics in NT1 at two European sleep centers (France, n = 381; Spain, n = 161) and measured IgG autoantibodies that recognize the GluN1 subunit of the NMDAR in 9 patients with NT1 with psychosis, and 25 NT1 patients without psychosis. Ten NT1 patients (6 in France, 4 in Spain) were diagnosed with comorbid psychosis, a frequency of 1.8%. One patient reported psychotic symptoms few months before narcolepsy onset, two patients few months after onset, and one patient one year after onset but after modafinil introduction. The six remaining patients reported long delays between NT1 and psychosis onset. Half the patients, mostly male adults, reported onset or worsening of psychotic symptoms after medication. We found no IgG antibodies to NR1/NR2B heteromers of the NMDARs in patients with NT1 with or without psychosis. To conclude, psychosis is rare in NT1, with limited evidence for a key impact of stimulants, and no association with anti-NMDAR antibodies. However, dramatic NT1 and schizophrenia exists especially in early onset NT1, which may lead to inappropriate diagnosis and management.

Narcolepsy type 1 (NT1) is a disabling orphan sleep disorder characterized by excessive daytime sleepiness and cataplexy. It is frequently associated with hypnagogic hallucinations and sleep paralysis, and is caused by hypocretin-1/orexin-A deficiency^1^. An autoimmune basis for NT1 has long been suspected based on its close association with the HLA DRB1^*^15:01-DQB1*06:02 haplotype, recent indirect evidence of an association between the T cell receptor alpha and the purinergic receptor P2RY11, epidemiological observations that H1N1 infection and vaccination are potential triggering factors, and the presence of elevated anti-tribbles homolog 2 and anti-streptolysin O antibodies^2^.

Psychiatric comorbidities are frequent in NT1, including mood, anxiety, attention deficit hyperactivity, and eating disorders, but rarely psychosis^3^,^4^,^5^,^6^,^7^,^8^. Both the frequency and underlying mechanisms of the association between NT1 and psychosis remain unclear. Previous studies have suggested that high-dose psychostimulants may induce psychosis in NT1 patients^6^,^7^. However, recent findings also suggest an overlapping autoimmune pathogenesis between NT1 and schizophrenia-like psychosis, associated with both HLA and autoantibodies^8^,^9^,^10^,^11^,^12^. For instance, prominent early-onset psychotic symptoms appeared in anti-N-methyl-D-aspartate receptor (NMDAR) encephalitis, a recently identified synaptic autoimmune disorder in which IgG autoantibodies recognize the glutamate (Glu) receptor type NMDA (NR1 subunit)^13^. In one study NMDAR autoantibodies were found in three of five patients with NT1 and severe psychosis^9^, but not in another population of ten patients affected with both NT1 and psychosis^7^.

The coexistence of NT1 and schizophrenia-like psychosis thus raises interesting pathophysiological questions about the potential role of an immune-mediated mechanism in the pathogenesis of psychotic symptoms in NT1. We decided to 1) estimate the frequency of schizophrenia-like psychosis and its characteristics in patients with NT1 at two large European sleep disorder centers; and 2) measure the presence of IgG autoantibodies that detect the GluN1 NMDAR subunit in this subpopulation and compare it with a group of patients with NT1 without psychosis.

## Results

From the two databases, a total of 542 patients were diagnosed with NT1, with only ten patients (six from Montpellier-France, four from Barcelona-Spain,) diagnosed with a comorbid schizophrenia-like psychosis, for an overall frequency of 1.8% (range 1.6–2.5%, depending on the sleep unit). Demographic and narcolepsy characteristics of the ten patients with NT1 comorbid with psychosis (six males, four females; mean age 32.8 ± 13.8) are summarized in Table 1. Narcolepsy started in childhood or adolescence (range 7–16 years) in seven patients (70%) while only 36% of patients from both cohorts started before 18 years. Overweight or obesity was detected in eight patients and significantly increased weight at narcolepsy onset (>10 kg in one year) in five patients. Clear-cut cataplexies were found with variable severity in all but one patient (with confirmed low CSF hypocretin-1 levels). Hallucinations and sleep paralysis were found in seven and six patients, respectively, at baseline. None of these patients had a family history of narcolepsy, previous H1N1 flu vaccination, or infection. One patient was mentally retarded. EEGs analyzed during PSG were normal, with no spike-and-wave discharge. MSLT latency and number of sleep onset REM periods confirmed the diagnosis when available (n = 9). All patients carried the HLA DQB1^*^06:02 allele. The eight patients with a lumbar puncture had low CSF hypocretin-1 levels (<110 pg/ml), with normal cell and protein levels.

Psychiatric assessment revealed paranoid delusions with disorganized thought and bizarre behavior associated with distress or disability in all ten patients. All patients had chronic psychosis with a formal diagnosis of schizophrenia-like psychosis according to DSM-IV-TR criteria, except for one patient (Case 9), who had a schizotypal personality disorder with two brief psychotic episodes triggered by both modafinil and methylphenidate use, and with no further episodes. Auditory hallucinations were found in all but one patient (Case 3), with magical thinking in one (Case 8). Aggressive behavior was reported in one patient, apathy in two, bulimia-like eating disorders during wakefulness in two, comorbid major depression in three, and drug abuse and dependence in two (heroin/cocaine and cannabis). Psychotic disorder severity differed across patients, with six requiring in-patient hospitalization at a psychiatric unit. No family history of psychosis or narcolepsy was found. Brain MRIs performed in eight patients were normal.

The onset of psychosis episodes varied between patients in terms of age, gender, and its relationships with narcolepsy onset and duration. Only one patient (a young female) reported psychotic symptoms a few months before narcolepsy onset, two drug-free young females a few months after sleepiness onset, and one adult male one year after narcolepsy onset but a few weeks after starting modafinil. The six remaining patients (all adults, 5 males) had long delays (10–24 years) between narcolepsy and psychosis onset. Triggers for the first psychotic episode were found in five patients: illicit drug use for two patients (cannabis and heroin/cocaine); and for three within the same time period, modafinil (600 mg/d) followed by methylphenidate (up to 120 mg/d– misuse), mazindol (3 mg/d), and sodium oxybate (9 g/d), respectively (Table 2). Two other patients reported worsening of psychotic symptoms after modafinil use (>300 mg daily) and sodium oxybate intake (9 g daily).

Both narcolepsy and psychosis management differed highly across patients. Daily use of psychostimulants (modafinil or pitolisant) at the time of study was reported by five patients, whereas anticataplectic drugs (fluoxetine, venlafaxine, or sodium oxybate) were reported by four patients (Table 1). Other drugs and various combinations of both psychostimulants and anticataplectics were prescribed but stopped due to ineffectiveness, poor compliance, or worsening of psychotic symptoms. Antipsychotic drugs (aripiprazole, risperidone, clozapine, olanzapine, ziprasidone) were prescribed to manage psychotic symptoms for seven patients, and two patients consumed methadone and hypnotic, respectively (for poor compliance with previously prescribed antipsychotic drugs) (Table 2). One patient (Case 9) who had only two brief psychotic episodes did not require antipsychotic drugs at the time of study.

We found no IgG antibodies against the NR1/NR2B heteromers of the NMDARs in the nine patients with NT1 and psychosis with either CSF (n = 2) or sera ( = 7) available (Table 2). These antibodies were also absent in the sera of the 25 NT1 patients without psychosis of similar age and gender.

## Discussion

In two large European cohorts of well-defined patients with NT1 (n = 542), we found 1) low frequency of comorbid schizophrenia-like psychosis (<2%), and mostly in adult patients with NT1 onset in infancy and adolescence, and 2) no associations with antibodies to NR1/NR2B heteromers of the NMDAR.

An association between psychosis and narcolepsy has been suggested in both adults and children. However, its frequency remains unknown, and results are highly variable between studies, ranging from 1% to 10%^3^,^6^,^7^,^8^,^9^. Due to some overlapping symptoms (e.g., hallucinations) and similar age of onset for both conditions, patients with schizophrenia may be misdiagnosed with narcolepsy, and patients with narcolepsy may be misdiagnosed with psychotic disorders^3^,^7^,^14^,^15^ Most patients with narcolepsy have sleep and body posture-related hallucinations, mainly visual or multisensory, and rarely unimodal verbal-auditory or stereotypic^3^,^7^,^14^,^15^,^16^,^17^. We found hypnagogic hallucinations in 70% of patients at disease onset, as expected in adults with NT1^1^,^3^,^14^, and various auditory stereotypic hallucinations in all patients but one at time of psychotic episode. We noted that 70% of the NT1-psychosis group had an early NT1 onset (below 16 years) compared to 35% of the non-psychosis NT1 patients. We also found widespread overweight/obesity (80% of patients), which was higher than expected in NT1 but previously described in children with NT1 comorbid with psychosis^1^,^8^. However, the higher weight in this subpopulation may be due to several mechanisms, such as NT1 *per se*, impulsive eating/bulima-like episodes, or antipsychotic drug use.

A recent study in a large cohort of children with NT1 showed that 10% later developed schizophrenia^8^. In most patients, psychotic symptoms developed within three years after narcolepsy onset, and often after psychostimulant intake. High-dose dopamine-induced psychostimulants (i.e., amphetamine, methylphenidate, and modafinil) as well as sodium oxybate may either aggravate positive psychotic symptoms (i.e., hallucinations and delusions) or trigger a psychotic episode in some predisposed NT1 patients^6^,^7^,^8^,^18^. Accordingly, in our clinical practice, we avoid prescribing high-dose medications (i.e., modafinil > 600 mg/day; methylphenidate > 60 mg/day; sodium oxybate > 9 g/day) for patients with narcolepsy to prevent such psychiatric side effects. A temporal connection with stimulant use was initially inferred in 50% of patients with narcolepsy who developed psychosis with high-dose psychostimulants, based on cessation of psychotic symptoms with reduction or discontinuation of the medications^6^. We found in the present study that only one patient fell into this group, and had no further psychotic symptoms in drug-free condition. However, half the patients (mostly male adults) reported either onset or worsening of psychotic symptoms after psychostimulant or sodium oxybate use. Nevertheless, two recent studies in adults and children with NT1 found that although narcolepsy drugs may trigger psychotic symptoms, withdrawal of either stimulants or antidepressants had no impact on psychotic symptoms *per se*^7^,^8^.

The management of narcolepsy symptoms therefore remains highly challenging: psychostimulants may be ineffective or may actually aggravate psychotic symptoms, whereas antipsychotic drugs may aggravate daytime sleepiness. On the other hand, we saw no changes in either the frequency or intensity of cataplexy under antipsychotic drugs. Several antidopaminergic antipsychotic drugs were prescribed for most of these patients, at different doses and with variable effectiveness on delusions, hallucinations, and disorganized thinking, which concurs with previous findings^7^,^8^. Anticataplectic drugs with serotonin-norepinephrine reuptake inhibitor properties were taken by 40% of patients to reduce cataplexy, hypnagogic/hypnopompic hallucinations, and sleep paralysis, with a good benefit/risk ratio. Overall, only half the targeted population was correctly managed for both narcolepsy and psychotic symptoms. This subpopulation often shows poor compliance with drug intake. Future pharmacological studies using non-dopaminergic stimulants such as the histamine H3 receptor inverse agonist (to avoid potential interaction with antipsychotic drugs^19^) are therefore needed to develop better management approaches for this severe comorbid condition.

Schizophrenia-like psychosis symptoms persisted in most NT1 patients (n = 9), suggesting a chronic association between the two conditions. However, the frequency of this association reached that expected for the general population (~1.5%)^20^. We also found substantial heterogeneity in the relationships between psychosis and age, gender, age at narcolepsy onset, psychostimulant triggers, and response to medication intake. Further examinations of blood, CSF, HLA typing, brain MRI, and EEG remained unremarkable, precluding definitive conclusions on the dysfunctions involved in this complex comorbid condition. However, the coexistence of NT1 and schizophrenia-like psychosis raises interesting pathophysiological questions. An autoimmune basis of schizophrenia was previously suggested^21^, and several studies have underscored its association with the HLA region, and particularly the DQB1*0602 subtype^11^,^12^. However, the latter association was weak compared to the largest HLA association yet reported between the DQB1*0602 subtype and NT1^1^,^22^. Only two studies focused on a potential autoimmune basis of the relationship between schizophrenia and NT1 in assessing NMDAR autoantibodies, typically found in either the serum or CSF of patients with anti-NMDA encephalitis. with discordant results obtained in NT1^8^,^9^. One study without estimate of the frequency of schizophrenia-like psychosis in NT1, reported the presence of NMDAR autoantibodies in 60% of patients with NT1 and severe psychosis, in 20% of patients with NT1 without psychotic symptoms, and in 8% of psychotic patients without encephalitis or sleep disorders^9^. We could not replicate these findings, as none of our nine patients with comorbid NT1 and psychosis and with available CSF/sera showed IgG NMDAR antibodies. These antibodies, assessed by rigorously validated method^23^, were also absent in the sera of the 25 NT1 patients without psychosis. These negative results corroborate a recent study of IgG NMDAR antibodies in 10 well-defined patients with NT1 and psychosis^7^.

Anti-NMDA encephalitis was recently clinically characterized as beginning with a viral prodrome followed by prominent psychotic symptoms (delusional thinking and hallucinations), insomnia rather than hypersomnia, and agitation, followed by neurological symptoms (confusion, seizures, severe dysautonomia) leading to central hypoventilation, which often requires intensive care management in the absence of early and aggressive immunosuppression^13^,^23^. This often fatal encephalitis is clinically recognizable, biologically diagnosable, and above all treatable with immunotherapy^13^. As described previously, clinical features of NT1 differed from anti-NMDA encephalitis, with neither cataplexy nor excessive daytime sleepiness at disease onset^24^, and none of our patients with NT1 comorbid with psychosis had seizures, epileptic discharges, or hypoventilation, or required intensive care. Taken together, our results point to a different pathophysiology between anti-NMDA encephalitis and the rare comorbid NT1 and psychosis. Based on our present findings, we could not recommend to use immune-based therapy to treat these rare patients with comorbid NT1 and schizophrenia-like psychosis.

This study includes certain limitations. First, the retrospective analysis of clinical data without formal psychiatric diagnostic measures may have affected both the frequency and diagnosis of psychotic disorders in well characterized NT1 patients. Nevertheless, detailed medical records were available to allow accurate diagnoses. A second limitation is that our clinical-based sample included mostly adult patients (>80%), and may not be fully representative of narcoleptic patients in the general population. Third, we have no control healthy group to assess the frequency of psychotic disorders in the general population to compare with the one obtained in our population of NT1.

In conclusion, based on two large European well-defined cohorts of adult patients with type 1 narcolepsy, we reported that schizophrenia-like psychosis is rarely comorbid with NT1(<2%), and with limited evidence for a key impact of psychostimulants. We found neither clinical association between anti-NMDA encephalitis and NT1, nor with IgG antibodies to NR1/NR2B heteromers of the NMDARs. However, dramatic NT1 can co-occur with schizophrenia especially in early onset NT1, which may delay diagnosis and/or lead to inappropriate diagnosis and management. Narcolepsy should be included in the differential diagnosis of refractory psychosis, especially in the presence of sleep-related and non-stereotypic hallucinations. Finally, the optimal management of this rare combination of psychotic and narcolepsy symptoms remains to be better defined.

## Methods

### Patients

We diagnosed 381 and 161 patients with NT1 at the National Reference Center for Narcolepsy, Sleep Unit, Gui-de-Chauliac Hospital, Montpellier-France between 1995 and 2014, and at the Multidisciplinary Sleep Disorders Unit, Hospital Clínic of Barcelona-Spain between 1988 and 2014, respectively. All patients met the International Classification of Sleep Disorders criteria for NT1 (history of definite cataplexy and mean daytime sleep latency ≤8 minutes with ≥2 sleep onset REM periods, or cerebrospinal hypocretin-1 deficiency when available)^25^. In the Montpellier cohort, 61% were males and the mean age at onset of narcolepsy was 23.97 ± 12.17 years (range 3–63) with 38.8% of patients with onset below 18 years. In the Barcelona cohort 59% were males, mean age at onset of narcolepsy 24.1 ± 12 years (range 5–56) with 32.9% with onset below 18 years.

All patients with a suspicion of associated psychotic disorder underwent a clinical face-to-face interview covering medical comorbidities, including an assessment to diagnose psychosis disorders according to DSM-IV-TR or previous version, depending on the assessment date^26^. Clinical history of narcolepsy, characteristics of psychotic symptoms, dose and duration of psychostimulant medications, and antipsychotic drug use were noted.

### Biological measurement

For most patients, DNA and frozen serum or cerebrospinal fluid (CSF) were obtained from biobanks at the two sleep units. HLA DQB1*06:02 typing was performed for all patients and CSF hypocretin-1 levels for 30.4% of the Barcelona group and 18.4% for the Montpellier group.

The nine patients with NT1 and psychosis and a non-selected group of twenty-five consecutive NT1 patients without psychosis followed at the Hospital Clínic of Barcelona (14 males and 11 females, mean age 32.6; SD:9.8; range 20–59 years) were tested for IgG NMDAR antibodies by immunofluorescence on HEK293 cells transfected with the NR1 and Nr2 subunits of the NMDAR receptor, as described previously^23^. Serum samples were tested at starting dilution of 1:40 and CSF undiluted (serum; n:7 NT1 with psychosis and all the 25 NT1 without psychosis; CSF; n:2 NT1 with psychosis). After 30 min, slides were washed with PBS-Tween for >5 min. Bound antibodies were labeled with fluorescein-conjugated goat anti-human IgG (DiaMed, Canton, OH; dilution 1:800) for 30 min.

**Ethics statement.** This study was approved by the institutional review boards of the University of Montpellier-France and the Hospital Clínic of Barcelona-Spain. The methods were carried out in accordance with the approved guidelines. Each participant signed legal consent forms. Informed consent was obtained from all subjects.

## Additional Information

**How to cite this article**: Dauvilliers, Y. *et al.* Absence of NMDA receptor antibodies in the rare association between Type 1 Narcolepsy and Psychosis. *Sci. Rep.* **6**, 25230; doi: 10.1038/srep25230 (2016).

## Figures and Tables

**Table 1:** Narcolepsy characteristics of 10 patients with narcolepsy type 1 comorbid with psychosis.

	Case 1 S-925 Spain	Case 2 NUR-055 Spain	Case 3 S-462 Spain	Case 4 S-412 Spain	Case 5 WG France	Case 6 CT France	Case 7 LG France	Case 8 MR France	Case 9 JM France	Case 10 NY France
Gender Male (M)/Female (F)	F	M	F	F	M	M	M	M	M	F
Age	16	34	38	20	40	48	59	22	30	21
BMI	31.3	34.9	36.1	46.8	28.3	28.9	39.7	22	27	22
Age at sleepiness onset	16	8	14	7	15	36	30	10	29	12
Age at cataplexy onset	16	10	na	8	26	42	33	15	29	12
Cataplexy frequency	1 per week	>1 per year and <1 per month	na	1 per month	1 per year	1 per week	>1 per day	>1 per day	>1 per day	>1 per year and <1 per month
Hypnagogic hallucinations	Yes	No	No	Yes	No	Yes	Yes	Yes	Yes	Yes
Sleep paralysis	Yes	Yes	No	No	No	Yes	Yes	Yes	Yes	No
MSLT latency, min	na	0.9	0.3	4.6	5.6	2	4.5	2.6	5.4	4.2
Number of SOREMPS, n	na	5	3	2	3	5	5	5	3	4
HLA DQB1	DQB1*06:02/03:01	DQB1*06:02/05:01	DQB1*06:02	DQB1*06:02	DQB1*06:02/03:0X	DQB1*06:02/06:02	DQB1*0602	DQB1*0602	DQB1*0602/0501	DQB1*0602
CSF hypocretin-1, pg/ml	88	na	<10	<10	39	<10	na	<10	<10	<10
Narcolepsy medication	Fluoxetine 40 mg/d	No drug	Modafinil 200 mg	No drug	No drug	Modafinil 400 mg/d Venlafaxine 75 mg/d	Modafinil 400 mg/d Venlafaxine 300 mg/d	Modafinil 300 mg/d Sodium Oxybate 7 g/d Venlafaxine 75 mg	Pitolisant 40 mg/d	No drug

**Table 2:** Characteristics of psychosis and measurements of IgG antibodies to the N-methyl-D-aspartate receptor (NMDAR) in 10 patients with narcolepsy type 1 comorbid with psychosis.

	Case 1 S-925 Spain	Case 2 NUR-055 Spain	Case 3 S-462 Spain	Case 4 S-412 Spain	Case 5 WG France	Case 6 CT France	Case 7 LG France	Case 8 MR France	Case 9 JM France	Case 10 NY France
Auditory hallucinations	Yes	Yes	No	Yes	Yes	Yes	Yes	Yes	Yes	Yes
Psychiatric hospitalization required	Yes, 24 days	Yes, 1 month	No	No	No	Yes, 2 weeks	Yes, several months	Yes, 3 weeks	No	Yes, several months
Age at onset of first psychosis episode	15 (a few months before narcolepsy)	27 (19 years after narcolepsy)	17 (at sleepiness onset)	7 (at sleepiness onset)	39 (24 years after narcolepsy)	47 (21 years after narcolepsy)	45 (12 years after narcolepsy)	23 (13 years after narcolepsy)	30 (1 year after sleepiness onset but few weeks after modafinil)	22 (10 years after narcolepsy)
Triggering factors	No	No	No But worsening after modafinil	No But worsening after sodium oxybate	Heroin and cocaine abuse	Sodium Oxybate	Mazindol	Cannabis	Modafinil and then methylphenidate	No
Medications for psychiatric symptoms	Clozapine 150 mg/d	Aripiprazole 30 mg/d	Risperidone 3 mg/d Ziprasidone 80 mg/d	Olanzapine 15 mg/d Fluoxetine 20 mg for compulsive eating	Methadone 130 mg/d	Alimemazine 15 mg/d	Olanzapine 15 mg/d	Aripiprazole 15 mg/d	No drugs	Aripiprazole 20 mg/d
IgG antibodies to NMDA receptor	Negative (CSF)	Negative (serum)	Negative (CSF)	Negative (serum)	Negative (serum)	Negative (serum)	Negative (serum)	Negative (serum)	Negative (serum)	na
